# Continuing Professional Development – Answers

**DOI:** 10.1002/jmrs.731

**Published:** 2023-12-12

Maximise your CPD by reading the selected article and answer the five questions. Please remember to self‐claim your CPD and retain your supporting evidence. Answers are available online at www.asmirt.org/news‐and‐publications/jmrs.

## Answers to Questions Published in Previous Issues

### Volume 69, Issue 4 December 2022

Castillo C, Steffens T, Livesay G, et al. IMPACT (Information Medically Pertinent in Acute Computed Tomography) requests: Delphi study to develop criteria standards for adequate clinical information in computed tomography requests in the Australian emergency department. *J Med Radiat Sci*. 2022 Dec; 69(4): 421–430. https://doi.org/10.1002/jmrs.607

CPD Questions https://doi.org/10.1002/jmrs.621

**Table:** 

Questions	Answers
1	C
2	B
3	C
4	C
5	B

Smith‐Lickess SK, Stefanic N, Shaw J, et al. What is the effect of a low literacy talking book on patient knowledge, anxiety and communication before radiation therapy starts? A pilot study. *J Med Radiat Sci*. 2022 Dec; 69(4): 463–472. https://doi.org/10.1002/jmrs.606

CPD Questions https://doi.org/10.1002/jmrs.628

**Table:** 

Questions	Answers
1	D
2	C
3	A
4	C
5	C

### Volume 70, Issue 1 March 2023

Pinson JA, King OA, Dennett AM, et al. Exploring the role of medical imaging assistants in Australian medical imaging departments: a mixed‐methods study. *J Med Radiat Sci*. 2023 Mar; 70(1): 46–55. https://doi.org/10.1002/jmrs.623

CPD Questions https://doi.org/10.1002/jmrs.644

**Table:** 

Questions	Answers
1	C
2	D
3	B
4	B
5	A

Mark F, Alnsour A, Penfold SN, et al. Volumetric modulated arc therapy (VMAT) comparison to 3D‐conformal technique in lung stereotactic ablative radiotherapy (SABR). *J Med Radiat Sci*. 2023 Mar; 70(1): 72–80. https://doi.org/10.1002/jmrs.634

CPD Questions https://doi.org/10.1002/jmrs.659

**Table:** 

Questions	Answers
1	D
2	D
3	C
4	B
5	A

### Volume 70, Special Issue 2, 2023

Phonlakrai M, Ramadan S, Simpson J, et al. Determination of hepatic extraction fraction with gadoxetate low‐temporal resolution DCE‐MRI‐based deconvolution analysis: validation with ALBI score and Child‐Pugh class. *J Med Radiat Sci*. 2023 Apr; 70(Suppl 2): 48–58. https://doi.org/10.1002/jmrs.617

CPD Questions https://doi.org/10.1002/jmrs.645

**Table:** 

Questions	Answers
1	D
2	A
3	A
4	B
5	D

Gibbons E, Hoffmann M, Westhuyzen J, et al. Clinical evaluation of deep learning and atlas‐based auto‐segmentation for critical organs at risk in radiation therapy. *J Med Radiat Sci*. 2023 Apr; 70(Suppl 2): 15–25. https://doi.org/10.1002/jmrs.618

CPD Questions https://doi.org/10.1002/jmrs.655

**Table:** 

Questions	Answers
1	B
2	C
3	D
4	A
5	D

### Volume 70, Issue 2 June 2023

Bantas G, Sweeney RJ, Mdletshe S. Digital radiography reject analysis: a comparison between two radiology departments in New Zealand. *J Med Radiat Sci*. 2023 Jun; 70(2): 137–144. doi: 10.1002/jmrs.654

CPD Questions doi: 10.1002/jmrs.679

**Table:** 

Questions	Answers
1	C
2	C
3	A
4	D
5	B

Laing B, Caldwell P, Vincent D, Rattray G. An evaluation of radiation therapy patient body mass index trends and potential impact on departmental resource planning. J Med Radiat Sci. 2023 Jun; 70(2):145–153. https://doi.org/10.1002/jmrs.652

CPD Questions https://doi.org/10.1002/jmrs.673

**Table:** 

Questions	Answers
1	B
2	B
3	C
4	C
5	C

### Volume 70, Issue 3 September 2023

Edwards C, Perry R, Chester D, Childs J. Entrustable professional activities of graduate accredited General Medical Sonographers in Australia – industry perceptions. *J Med Radiat Sci*. 2023 Sep; 70(3): 229–238. https://doi.org/10.1002/jmrs.676

CPD Questions https://doi.org/10.1002/jmrs.696

**Table:** 

Questions	Answers
1	D
2	B
3	A
4	C
5	B

Fong SC, Pandey R, Rajaretnam M, Delaibatiki M, Peel DNY. Routine prophylactic percutaneous endoscopic gastrostomy in head and neck cancers with bilateral neck irradiation: a regional cancer experience in New Zealand. *J Med Radiat Sci*. 2023 Sep; 70(3): 292–300. https://doi.org/10.1002/jmrs.699

CPD Questions https://doi.org/10.1002/jmrs.708

**Table:** 

Questions	Answers
1	C
2	B
3	C
4	A
5	B

